# Addressing Latent Tuberculosis Infection Treatment Through a Collaborative Care Model With Community Pharmacies and a Health Department

**DOI:** 10.5888/pcd17.190263

**Published:** 2020-02-13

**Authors:** Bernadette Jakeman, Stefanie J. Logothetis, Melissa H. Roberts, Amy Bachyrycz, Diana Fortune, Matthew E. Borrego, Julianna Ferreira, Marcos Burgos

**Affiliations:** 1 University of New Mexico College of Pharmacy, Department of Pharmacy Practice and Administrative Sciences, Albuquerque, New Mexico; 2 New Mexico Department of Health, Tuberculosis Program, Santa Fe, New Mexico; 3 University of New Mexico School of Medicine, Division of Infectious Diseases, Albuquerque, New Mexico; 4 New Mexico Veterans Affairs Health Care System, Albuquerque, New Mexico

## Abstract

**Introduction:**

The objective of this study was to evaluate a novel collaborative care model using community pharmacies as additional access points for latent tuberculosis infection (LTBI) treatment for patients using combination weekly therapy with isoniazid and rifapentine (3HP) plus directly observed therapy for 12 weeks.

**Methods:**

This prospective pilot study included adult patients diagnosed with LTBI. Patients were eligible for study participation if they spoke English or Spanish and were followed by the New Mexico Department of Health (NM DOH). Patients were excluded if they were pregnant, receiving concomitant HIV antiretroviral therapy, or had contraindications to 3HP due to allergy or drug interactions. Community pharmacy sites included chain, independent, and hospital outpatient pharmacies in Albuquerque and Santa Fe, New Mexico.

**Results:**

A total of 40 patients initiated treatment with 3HP and were included. Most were female (55%) and had a mean age of 46 years (standard deviation, 12.6 y). A total of 75.0% of patients completed LTBI treatment with 3HP in a community pharmacy site. Individuals of Hispanic ethnicity were more likely to complete treatment (76.7% vs 40.0%, *P* = .04). Most patients (60%; n = 24) reported experiencing an adverse drug event (ADE) with 3HP therapy. Patients who completed treatment were less likely to experience an ADE than patients who discontinued treatment (50.0% vs 90.0%, *P* = .03). Pharmacists performed 398 LTBI treatment visits (40 initial visits, 358 follow-up visits), saving the NM DOH approximately 143 hours in patient contact time.

**Conclusion:**

High completion rates and safe administration of LTBI treatment can be achieved in the community pharmacy setting.

**Summary**

**What is already known on this topic?**

It is estimated that 13 million people in the United States have latent tuberculosis infection (LTBI). This large number of potential LTBI cases poses a challenge for successful tuberculosis control and elimination.

**What is added by this report?**

We examined a novel, collaborative care model using community pharmacies as additional access points for LTBI treatment for patients using combination weekly therapy with isoniazid and rifapentine and directly observed therapy for 12 weeks.

**What are the implications for public health practice?**

High completion rates and safe administration of LTBI treatment can be achieved in the community pharmacy setting.

## Introduction

Tuberculosis (TB) is a curable disease, yet it is the tenth leading cause of death worldwide, ranking above HIV (1). TB disease resulted in an estimated 1.3 million deaths worldwide in 2017 (1). The World Health Organization has outlined a framework for TB elimination in low-incidence countries such as the United States (2). Included in the TB elimination strategy is the identification and treatment of latent tuberculosis infection (LTBI) to prevent progression to and transmission of active disease (2). Treatment of LTBI decreases illness and death associated with active TB disease (3) and is associated with less medication toxicity and cost compared with active TB disease treatment (4–6). It is estimated that 13 million people have LTBI in the United States (7). This large number of potential LTBI cases poses a serious public health challenge for successful TB control and elimination. Using community pharmacies is a possible strategy to expand access for testing and treatment.

In 2011, because of nursing resource limitations at the New Mexico Department of Health (NM DOH), tuberculin skin testing was made available in New Mexico community pharmacies (8). As of 2016, more than 200 New Mexico pharmacists had been trained to provide this public health service, which provides testing access for patients in small city locations and has been widely used by patients across the state (8,9). In 2017, the NM DOH TB program wanted to expand access to patients by also providing LTBI treatment in the community pharmacy setting.

In 2012 the NM DOH transitioned from LTBI treatment with isoniazid monotherapy to weekly combination therapy with isoniazid plus rifapentine (3HP). This short 12-week combination regimen is associated with higher completion rates and lower rates of hepatotoxicity (10,11). However, the 3HP regimen is still associated with medication toxicity, drug–drug interactions, and nonadherence. Providing this once-weekly regimen in a community pharmacy setting is one potential option to address these issues.

Completion rates for LTBI vary considerably in the literature, ranging from 35%–90%, with higher completion rates generally reported with shorter treatment regimens (10,12–17). Predictors for noncompletion include unstable housing, tobacco use, alcohol use, adverse drug events (ADEs), older age, patient location, poverty, and non-Hispanic ethnicity (14,18–21).

Data evaluating the use of pharmacists in the treatment of LTBI are limited (22–25). However, the available studies have reported high completion rates when a pharmacist was included in treatment management. Tavitian et al reported high completion rates (93%) associated with a pharmacist-managed clinic for treatment of LTBI with isoniazid monotherapy in health care workers (22). Carter et al also reported high LTBI completion rates (94%) with a pharmacist-run clinic using monotherapy with either rifampin or isoniazid for refugee patients (25). To our knowledge, administration of LTBI treatment in the community pharmacy setting has never been evaluated. 

The primary objective of this study was to evaluate a novel and collaborative care model using community pharmacy sites to support increased patient access to LTBI treatment using combination weekly therapy with isoniazid 900 mg plus rifapentine 900 mg for 12 weeks. Secondary objectives were evaluation of treatment completion rates and ADEs.

## Methods

The University of New Mexico Health Sciences Research Protection Office institutional review board approved the study protocol. This prospective pilot study included adult patients ≥18 years of age who were diagnosed with LTBI by a physician at the NM DOH. Patients were eligible for study participation if they spoke English or Spanish and were followed by the NM DOH offices in Albuquerque or Santa Fe, New Mexico. Patients also had to be able to take LTBI treatment with weekly combination therapy with 900 mg of rifapentine and 900 mg of isoniazid for 12 weeks. Patients were excluded if they were pregnant, receiving concomitant HIV antiretroviral therapy, or had contraindications to 3HP due to allergy or drug interaction. Eligible patients with newly diagnosed LTBI who were seen at the Santa Fe or Albuquerque departments of health from February 2017 through April 2018 were given the choice to receive usual care through the NM DOH or participate in the study, receiving 3HP with directly observed therapy (DOT) at a participating community pharmacy of their choice. 

Before consent, patients were provided with a list of 9 possible pharmacy locations (3 pharmacies in Santa Fe and 6 pharmacies in Albuquerque) and their hours of operation. Study investigators identified and contacted 10 pharmacies as potential pilot sites based on geographical distribution and site diversity. Nine pharmacies agreed to participate. Community pharmacy sites included chain, independent, and hospital outpatient pharmacies, in Albuquerque and Santa Fe, New Mexico (NM). The pharmacies were geographically distributed throughout the cities to provide a variety of pharmacy locations for study participants. Patients were allowed to choose only 1 pharmacy location and could not switch locations after consent. No study incentive was offered to patients. Medications were provided to patients at no charge regardless of study participation. Women of childbearing age were counseled to use a barrier birth control method before enrollment and with rifapentine initiation. Study investigators consented patients for study participation at the NM DOH clinics. Baseline laboratory tests (liver function tests, complete blood counts, comprehensive metabolic panel, and HIV with opt-out option) were drawn at the NM DOH before 3HP initiation. Patient demographics, comorbidities, and additional TB risk factors (1) were collected to characterize the patient population being served.

Before implementation, participating pharmacies (range, 1–3 pharmacists per pharmacy) attended (either in person or via videoconferencing) a 2-hour accredited continuing education training on LTBI treatment held at the University of New Mexico. NM DOH nursing personnel at the participating department of health locations were also included in the training program. The NM DOH TB program medical director, the NM DOH TB program manager/TB nurse consultant, and an infectious diseases pharmacist provided the training for community pharmacies. The infectious diseases pharmacist trained additional pharmacists who joined the project at a later date, using the same training materials.

After consenting a patient for study participation, the patient’s prescriptions for rifapentine 900 mg (4 tablets) and isoniazid 900 mg (3 tablets), to be taken weekly with DOT, were faxed to the participating pharmacy with 11 refills (12 doses total of 3HP). The TB physician could adjust the dose for weight. The TB physician could also order pyridoxine (vitamin B6) if appropriate.

The participating pharmacies were responsible for acquiring and storing the LTBI medications according to the state law and pharmacy policy. The cost of the medication varied for each site (~$25–30/week). Grant funds provided pharmacies adequate compensation to cover the cost of the medication and pharmacist time. In addition, telephonic interpreter services, also provided through grant funding, were available for Spanish-speaking patients at all pharmacy locations.

Participating pharmacies followed the NM DOH nursing protocol for LTBI treatment with DOT. Patients picked up their weekly doses at the community pharmacy. At each visit the pharmacist would 1) complete a drug–drug interaction evaluation, 2) screen the patient for 3HP treatment toxicity, 3) screen the patient for symptoms of active TB disease, 4) provide the LTBI treatment medication, and 5) watch the patient take the medication (DOT). Screening questions were adopted from the DOH LTBI treatment protocol. If a patient developed signs and symptoms suggestive of liver or hematologic toxicity, the pharmacist contacted the DOH TB program nurse manager and instructed the patient to hold the medications. Potentially serious ADEs were reported to the NM DOH TB Program and reviewed by the TB physician. Reportable potential ADEs were jaundice, persistent nausea or vomiting, abdominal pain, easy bruising or bleeding, and changes in urine or stool color. Medications could be resumed or discontinued after evaluation by the NM DOH TB program’s medical director. Patients could discontinue treatment at the community pharmacy and complete treatment through the NM DOH if closer follow-up was required by the TB physician. Treatment was considered complete if patients received 12 doses. To be consistent with DOH completion rate reporting calculations, patients who did not start therapy were not included in the data analysis.

Continuous variables were described by using measures of central tendency (mean, standard deviation [SD]), and binary and categorical variables were described by using the number of nonmissing and missing observations and the frequency and percentage of responses. Patients receiving treatment were categorized into 2 groups: 1) those receiving the complete 12 doses of treatment and 2) those receiving partial treatment. Statistical differences between the 2 groups were determined using the Student’s *t* test for continuous variables (pooled method for equal variances, Satterthwaite method for unequal variances) and Fisher exact test for binary and categorical variables. All tests were 2-sided and used a significance level of *P* < .05. SAS statistical software version 9.4 (SAS Institute, Inc) was used to perform analyses.

## Results

Of the 41 patients who consented to participate in the study during the evaluation period, 40 initiated treatment and were included in the data analysis (Figure). Thirty patients received LTBI treatment at an Albuquerque community pharmacy, and 10 patients received LTBI treatment at a Santa Fe community pharmacy. Most patients were female (55%; n = 22), Hispanic white (37.5%; n = 15), and had an average age of 46 years (SD, 12.6 y) (Table 1).

**Figure:**
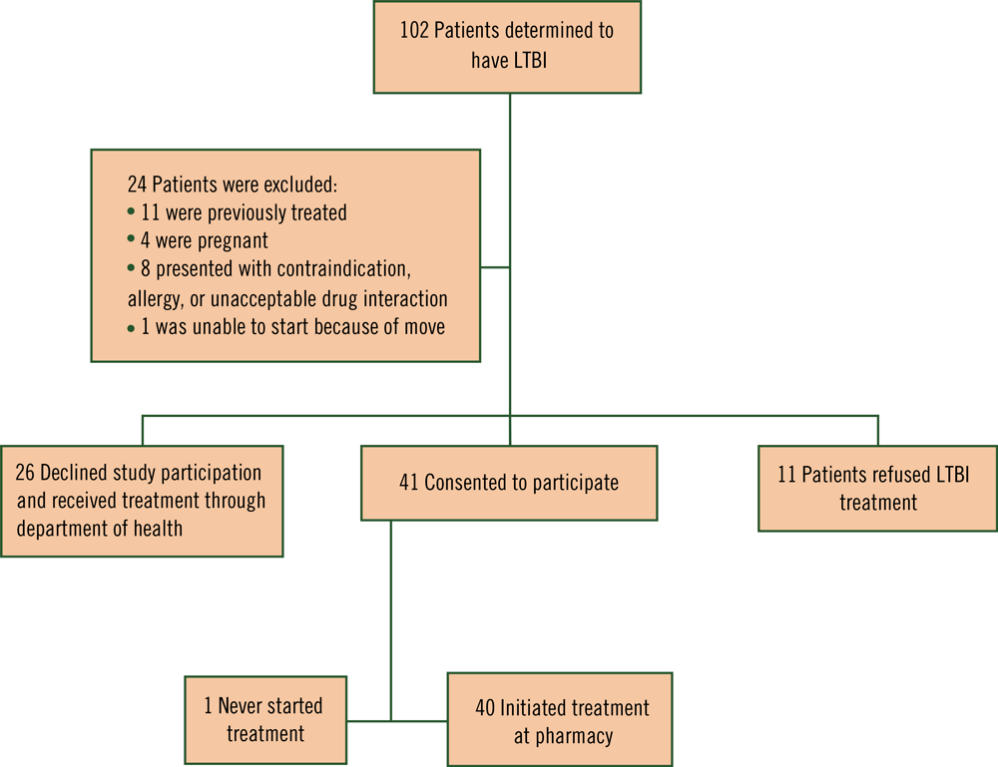
Flow diagram for patient enrollment, study on using a collaborative care model to treat LTBI, New Mexico, 2017–2018. Abbreviation: LTBI, latent tuberculosis infection.

**Table 1:** Treatment Completion Rates of Participants (N = 40), by Demographic Characteristics, Study on Using a Collaborative Care Model to Treat LTBI, New Mexico, 2017–2018

Demographic Characteristic	Value^a^	Completed Treatment^b^ (n = 30)	Discontinued Treatment (n = 10)	*P* Value^b^
**Male sex**	18 (45.0)	15 (50)	3 (30)	.46
**Mean age, y (SD)**	46.0 (12.6)	45.6 (14.0)	47.2 (7.5)	.73
**Mean BMI, kg/m^2^ (SD)**	28.6 (6.1)	28.0 (5.6)	30.2 (7.5)	.37
**Hispanic ethnicity**				
Yes	27 (67.5)	23 (76.7)	4 (40)	.04
No	12 (30.0)	7 (23.3)	5 (50)	
Unknown	1 (2.5)	0	1 (10)	
**Race/ethnicity**				
Hispanic white	15 (37.5)	12 (40.0)	3 (30)	.008
Hispanic other	12 (30.0)	11 (36.7)	1 (10)	
Non-Hispanic white	7 (17.5)	6 (20.0)	1 (10)	
Non-Hispanic black	2 (5.0)	1 (3.3)	1 (10)	
Non-Hispanic Asian	3 (7.5)	0	3 (30)	
Unknown	1 (2.5)	0	1 (10)	
**Birth country**				
United States	10 (25.0)	7 (23.3)	3 (30)	.70
Non-US	29 (72.5)	22 (73.3)	7 (70)	
Unknown	1 (2.5)	1 (3.3)	0	
**Substance use**				
Alcohol	10 (25.0)	7 (23.3)	3 (30)	.69
Tobacco	14 (35.0)	12 (40.0)	2 (20)	.45
**Comorbidities^b^**				
Diabetes	7 (17.5)	6 (20.0)	1 (10)	.66
Asthma	2 (5.0)	2 (6.7)	0	>.99
End-stage renal disease	4 (10.0)	4 (13.3)	0	.56

Abbreviations: BMI, body mass index; LTBI, latent tuberculosis infection; SD, standard deviation.

a Values are no. (%) unless otherwise indicated.

b Determined using *t* test for mean differences and Fisher exact test for frequency differences.

Of 40 patients who initiated treatment, 75% (n = 30) completed LTBI treatment with 3HP at 1 of the participating community pharmacy sites. Seven patients discontinued 3HP because of potential ADEs, and 3 patients were lost to follow-up. A higher percentage of patients who completed treatment were of Hispanic ethnicity compared with patients who discontinued treatment (76.7% vs 40.0%, *P* = .04) (Table 1). Other demographic characteristics, including age, sex, and substance use (ie, tobacco or alcohol) did not differ between patients who completed or discontinued LTBI treatment. Most patients (60%; n = 24) reported experiencing an ADE with 3HP therapy (Table 2). The most common ADEs reported were dark urine (27.5%; n = 11), excessive fatigue (22.5%; n = 9), and nausea/vomiting (22.5%; n = 9). Differences between the groups were significant with regard to ADEs. Fewer patients who completed treatment experienced any ADE compared with patients who discontinued treatment (50% vs 90%, *P* = .03). ADEs that patients who completed treatment experienced less often than those who discontinued treatment were excessive fatigue (13.3% vs 50.0%, *P* = .03) and nausea/vomiting (13.3% vs 50.0%, *P* = .03). Potentially serious ADEs were reported to the NM DOH TB Program and reviewed by the TB physician. In 7 cases (17.1%) it was determined that the patient should discontinue 3HP treatment. Of the 7 patients who discontinued 3HP therapy at a community pharmacy site, 1 was able to complete LTBI therapy with another LTBI regimen through the NM DOH, bringing the overall completion rate to 77.5%. No cases of active tuberculosis or death were reported during the study period. The average number of doses received by patients who discontinued therapy at a community pharmacy was 3.8 (SD, 2.3).

**Table 2:** Reported Adverse Drug Events of Patients (N = 40), by Patient Treatment Completion Status, Study on Using a Collaborative Care Model to Treat LTBI, New Mexico, 2017–2018

Type of ADE	No. (%)	Completed Treatment^a^ (n = 30)	Discontinued Treatment (n = 10)	*P* Value^a^
		No. (%)		
Any	24 (60.0)	15 (50.0)	9 (90)	.03
Dark urine	11 (27.5)	8 (26.7)	3 (30)	>.99
Nausea/vomiting	9 (22.5)	4 (13.3)	5 (50)	.03
Excessive fatigue	9 (22.5)	4 (13.3)	5 (50)	.03
Appetite loss	6 (15.0)	3 (10.0)	3 (30)	.15
Abdominal discomfort	6 (15.0)	3 (10.0)	3 (30)	.15
Flu-like symptoms	5 (12.5)	2 (6.7)	3 (30)	.09
Urine output change	3 (7.5)	1 (3.3)	2 (20)	.15
Stool color change	3 (7.5)	1 (3.3)	2 (20)	.15
Rash/itching	2 (5.0)	0	2 (20)	.06
Numbness or tingling	2 (5.0)	2 (6.7)	0	>.99
Fever >3 days	1 (2.5)	1 (3.3)	0	>.99
Jaundice	0	0	0	>.99
Bleeding/bruising	0	0	0	>.99
Other	13 (32.5)	10 (33.3)	3 (30)	>.99
**Number of ADEs**				
0	16 (40.0)	15 (50.0)	1 (10)	.03
1	8 (20.0)	6 (20.0)	2 (20)	
2	7 (17.5)	4 (13.3)	3 (30)	
3	1 (2.5)	1 (3.3)	0	
4	1 (2.5)	0	1 (10)	
5	3 (7.5)	2 (6.7)	1 (10)	
6	2 (5.0)	2 (6.7)	0	
7	2 (5.0)	0	2 (20)	

Abbreviations: ADE, adverse drug event; LTBI, latent tuberculosis infection.

a Determined using *t* test for mean differences and Fisher exact test for frequency differences.

Pharmacists performed 398 LTBI treatment visits (40 initial visits, 358 follow-up visits) during the evaluation period. Pharmacists recorded the estimated time for initial and follow-up visits for 26 patients. The average time for an initial visit was 25 (SD, 10.1) minutes. The average time for follow up visits was 22 (SD, 9.7) minutes. The initiative saved the NM DOH more than 8,876 minutes (148 hours) in patient visit time. Most patients (62.5%; n = 25) lived 5 miles or less from the pharmacy where they received 3HP treatment (Table 3).

**Table 3:** Characteristics of Patient (N = 40) Pharmacy Visits, by Patient Treatment Completion Status, Study on Using a Collaborative Care Model to Treat LTBI, New Mexico, 2017–2018

Pharmacy Visit Characteristic	Value	Completed Treatment^a^ (n = 30)	Discontinued Treatment (n = 10)	*P* Value^a^
**Mean distance, mi (SD)**	5.2 (3.1)	5.4 (3.4)	4.8 (1.8)	.53
**Distance to pharmacy, mi**				
≤5	25 (62.5)	18 (60.0)	7 (70)	.79
>5	14 (35.0)	11 (36.7)	3 (30)	
Unknown	1 (2.5)	1 (3.3)	0	
**Days between positive test and treatment start**				
0–60	16 (40.0)	10 (33.3)	6 (60)	.55
61–120	14 (35.0)	11 (36.7)	3 (30)	
121–180	3 (7.5)	3 (10.0)	0	
>180	4 (10.0)	4 (13.3)	0	
Unknown	3 (7.5)	2 (6.7)	1 (10)	
**Initial visit time, min (SD) (n = 26) **	25.0 (10.1)	25.3 (9.3)	24.4 (12.1)	.84
**Follow up visit time, min (SD) (n = 25) **	22.0 (9.7)	23.75 (10.1)	18.9 (8.6)	.24

Abbreviations: ADE, adverse drug event; LTBI, latent tuberculosis infection; SD, standard deviation.

a Determined using *t* test for mean differences and Fisher exact test for frequency differences. Values are no. (%) unless otherwise indicated.

## Discussion

This is the first study to evaluate the feasibility of providing LTBI treatment with DOT in a community pharmacy setting as a strategy to improve patient access in collaboration with a state health department. We demonstrated that 3HP can be safely administered in a community pharmacy collaborative care setting and result in high rates of LTBI treatment completion (75% in community pharmacy setting; 77.5% overall). Our LTBI completion rate was similar to rates reported by the NM DOH. In 2017, the NM DOH reported that 374 patients in NM were determined to have LTBI at a DOH clinic location (26); 167 patients initiated treatment, 107 (64.1%) completed LTBI treatment (New Mexico Department of Health Tuberculosis Prevention Program, 2017, unpublished data). High rates of completion in the community pharmacy setting are likely a result of a variety of accessible pharmacy locations, extended operating hours, and no requirement for scheduled appointments.

In our cohort we found that Hispanic patients were more likely to complete LTBI treatment compared with non-Hispanics. This finding is consistent with those of prior studies (14,19–21,28,29), but its cause is unclear. However, perception of disease risk has been previously reported as a predictor for LTBI treatment completion (30), which this study did not assess. These patients may have had an increased perception of risk if they or their family members were born in TB-endemic areas.

We reported high rates of potential ADEs (n = 24; 60%). ADEs, including nausea/vomiting and fatigue, were associated with noncompletion of LTBI treatment in this study. This finding is also consistent with prior studies (18,21). Most potential medication side effects reported with 3HP were not serious and may have been due to other causes. Most patients were managed through the pharmacy with direct communication and collaboration with the DOH. This resulted in treatment completion in 17 of the 24 patients (71%) that experienced a potential ADE. Pharmacists were able to ensure pyridoxine (vitamin B6) supplementation when appropriate and discuss options to address nausea. Addressing potential ADEs in a community pharmacy setting is an opportunity to increase completion rates before losing patients to follow-up.

WHO describes the importance of accessible and free TB services in their elimination framework (2). Community pharmacies may be able to offer an additional accessible setting to provide LTBI treatment. To achieve TB elimination, it is important to increase access to treatment in ways that are convenient for patients while also relieving a portion of the burden from the DOH. A total of 41 of the 67 patients who received treatment chose to participate in this study and receive treatment at a community pharmacy site, highlighting patient interest in this treatment setting. With an increase in available community pharmacy sites, it is likely that more patients will have the opportunity to complete treatment.

Data from this pilot project provide important information about LTBI treatment administered in the community pharmacy setting. However, our study has limitations. First, results cannot be generalized outside of New Mexico. This was a pilot study with a small sample in 2 large city settings in New Mexico, which is largely rural and has health care professional shortages and patient socioeconomic barriers. In addition, only a small subset of the total number of pharmacies in the state participated in the study. To minimize this limitation, we included pharmacies that were geographically distributed, including both independent and chain pharmacies. We would not expect the study results to differ significantly if all New Mexico pharmacies offering this public health service had participated. In addition, we only evaluated LTBI treatment with 3HP plus DOT. Another consideration is that this project was supported by a grant, which covered the cost of 3HP medication provided in the community pharmacy. If this public health service is to be sustainably offered to patients at no charge, a mechanism will need to be identified to address the cost of providing this service in the community pharmacy setting. Finally, the success of this program can be attributed to the collaboration with the DOH, in which training and expert consultation was provided.

LTBI treatment with 3HP plus DOT can be safely administered in a community pharmacy collaborative care setting and offers opportunity for improved access to care for patients. High rates of completion in a community pharmacy setting are likely a result of increased access to care through neighborhood pharmacy locations, extended operating hours, and no requirement for scheduled appointments. The largest barrier to LTBI treatment completion was ADEs. Pharmacists can help identify and manage potential ADEs in the community pharmacy setting, which may minimize loss to follow-up.
